# Association between myocardial layer-specific strain and high 10-year risk of atherosclerotic cardiovascular disease in hypertension—findings from the China-PAR project study

**DOI:** 10.3389/fcvm.2024.1460826

**Published:** 2024-10-03

**Authors:** Jianxiong Chen, Xiaohuan Yang, Xinyi Li, Lin Jin, Lingheng Wu, Mengjiao Zhang, Lianfang Du, Xianghong Luo, Zhaojun Li

**Affiliations:** ^1^ Department of Ultrasound, Mindong Hospital Affiliated to Fujian Medical University, Ningde, Fujian, China; ^2^ Department of Ultrasound, Shanghai General Hospital of Nanjing Medical University, Shanghai, China; ^3^ Department of Ultrasound, Shanghai Fourth People’s Hospital Affiliated to Tongji University, Shanghai, China; ^4^ Business School, Hubei University, Wuhan, China; ^5^ Department of Ultrasound, Guanghua Hospital Affiliated to Shanghai University of Traditional Chinese Medicine, Shanghai, China; ^6^ Department of Medical Imaging, Weifang Medical University, Weifang, China; ^7^ Department of Ultrasound, Shanghai General Hospital, Shanghai Jiaotong University School of Medicine, Shanghai, China; ^8^ Department of Echocardiology, Shanghai General Hospital, Shanghai Jiaotong University School of Medicine, Shanghai, China

**Keywords:** echocardiography, hypertension, layer-specific longitudinal strain, layer-specific circumferential strain, two-dimensional speckle-tracking imaging

## Abstract

**Objectives:**

Myocardial layer-specific strain is a sensitive tool for detecting myocardial dysfunction. The objective of this study was to assess changes in the left ventricle (LV) function using myocardial layer-specific strain and its association with 10-year atherosclerotic cardiovascular disease risk (10Y-ASCVDR) in individuals with hypertension (HP).

**Methods:**

The parameters of LV structure, including layer-specific global longitudinal strain (GLS_ww_, GLS_endo_, GLS_mid_, GLS_epi_) and layer-specific global circumferential strain (GCS_ww_, GCS_endo_, GCS_mid_, GCS_epi_), were analyzed by two-dimensional speckle-tracking echocardiography in 239 hypertensive patients and 124 control subjects. In addition, participants were divided into low-risk (LR) and high-risk (HR) subgroups according to 10Y-ASCVDR scores . The correlation between myocardial layer-specific strain and 10Y-ASCVDR was further analyzed by the restricted cubic spline (RCS) function.

**Results:**

The values of GLS_ww_, GLS_epi_, GLS_mid_, and GLS_endo_ were significantly lower in HP patients with HR than in HP patients with LR and controls (*p <* 0.05). However, no significant differences in layer-specific GCS were observed between the groups (*p* > 0.05). RCS analysis revealed that 10Y-ASCVDR exhibited a significant J-shaped relationship with layer-specific GLS and GCS. After adjusting for confounding factors, GLS_ww_ (*β =* 0.156, *p =* 0.042), GLS_mid_ (*β =* 0.161, *p =* 0.032), GCS_endo_ (*β =* 0.163, *p =* 0.024), and GCS_mid_ (*β* = −0.175, *p =* 0.030) were identified as independent influencing factors for high 10Y-ASCVDR.

**Conclusions:**

In hypertensive patients, myocardial layer-specific strain, especially GLS, sensitively detected LV dysfunction and showed a significant J-shaped relationship with 10Y-ASCVDR. GCS_mid_ may have a compensatory effect on myocardial impairment. LV myocardial layer-specific strain may help to understand the early compensatory mechanisms of the myocardium in hypertension.

## 1 Introduction

The prevalence of hypertension has been steadily increasing annually. In China, the prevalence of hypertension among individuals aged 18 years and older has reached 27.9%, affecting over 200 million people, ranking first in the world (1, 2). Cardiovascular morbidity and mortality rates in hypertensive patients increase every year and can be two to four times higher than in non-hypertensive individuals (3). Accordingly, the medical community agrees that early detection and intervention are crucial for the prevention and treatment of cardiovascular complications in hypertensive patients (4).

Global circumferential strain (GCS) and global longitudinal strain (GLS) can be quantitatively assessed using two-dimensional speckle-tracking echocardiography (2D-STE) and are widely used in the clinic to evaluate overall and local cardiac function (5, 6). Previous studies have found that GLS is precociously altered early, even in the very early stages of hypertension, whereas GCS tends to be compromised in more advanced stages of arterial hypertension, especially when left ventricle (LV) hypertrophy has already been established (7, 8). The left ventricular wall comprises three different layers of muscle fibers (9). Previous reports have shown that whole-wall global longitudinal strain (GLS_ww_) and epimyocardial global longitudinal strain (GLS_epi_) have independent prognostic value in predicting cardiovascular events (9). Thus, we speculated that layer-specific GLS or GCS measured by 2D-STE might differ in individuals with high- and low-risk cardiovascular disease (CVD), with GLS changes possibly preceding GCS alterations in patients with hypertension.

Liu et al. (10) developed the Prediction for Atherosclerotic Cardiovascular Disease Risk in China (China-PAR) model. With a sample size of more than 127,000 individuals and a maximum follow-up period of more than 23 years, the model was designed to better predict the likelihood of an atherosclerotic cardiovascular disease (ASCVD) event in Chinese adults over the next 10 years (11, 12). Due to the lack of clinical outcome events, we chose the China-PAR model to predict subsequent CVD events in our cross-sectional study. However, the effectiveness of layer-specific GCS and GLS parameters in discriminating myocardial function in hypertensive patients with varying 10-year ASCVD risk remains unclear.

Therefore, this study aimed to investigate the relationship between myocardial layer-specific strain, including GLS and GCS, and 10-year atherosclerotic cardiovascular disease risk (10Y-ASCVDR) using the China-PAR model as the endpoint event in patients with hypertension.

## 2 Methods

### 2.1 Study population

From October 2020 to October 2023, a total of 253 hypertensive patients and 124 control participants from the Jiading Branch of Shanghai General Hospital, Shanghai, China, were enrolled in this cross-sectional observational study. The control group consisted of individuals who had undergone myocardial strain testing using the same protocol at our hospital and had no history of hypertension. They were selected based on their similar age distribution to the hypertensive group.

### 2.2 Inclusion and exclusion criteria

All patients met the following criteria: age 18–80 years; hypertension according to the 2020 International Society of Hypertension guidelines (13); and LV ejection fraction (LVEF) greater than 55%. The exclusion criteria were as follows: (1) diabetes; (2) coronary artery disease and abnormalities in ECG or other imaging examinations, which are known to be associated with coronary artery disease; (3) congestive heart failure; (4) secondary hypertension; (5) congenital heart disease; (6) non-sinus rhythms, such as atrial fibrillation or pacemaker rhythms; (7) previous stroke; (8) active cancer; (9) heart valve disease; and (10) myocardial disease.

### 2.3 Baseline measurements

The study participants completed a comprehensive health and lifestyle questionnaire, as well as a self-administered questionnaire, collecting information about their age, sex, smoking habits, history of diabetes, hypertension, and antihypertension drug therapy (including angiotensin-converting enzyme inhibitors, angiotensin receptor blockers, β-blockers, calcium channel blockers, and/or diuretics). Smokers were defined as those who had smoked ≥100 cigarettes in their lifetime (14). Body mass index (BMI) was calculated as weight/height^2^ (kg/m^2^), with nurses following standardized protocols for weight and height measurements.

### 2.4 Blood pressure measurement

Before the measurement, subjects rested for 5–10 min and were advised to stop smoking and avoid caffeine for at least 24 h. Measurements were taken while subjects were seated in a quiet, temperature-controlled room (24–26°C). Trained technicians used the AVE-2000Pro (PASESA, Tokyo, Japan) to measure SBP and DBP (15). Subjects were measured again after 2 min. The final results were derived from the average of the three readings.

### 2.5 Prediction of 10-year ASCVD risk

Using a specific questionnaire developed by the China-PAR model (16), general patient information and medical history were collected to calculate the 10Y-ASCVDR risk in people without prior ASCVD and to assess the likelihood of developing a first ASCVD event, including non-fatal myocardial infarction, coronary heart disease death, or fatal/non-fatal stroke. The 10Y-ASCVDR prediction can estimate the risk of developing an ASCVD event for the first time (11). In the China-PAR model, the following variables are included: sex, age, total cholesterol (TC), high-density lipoprotein cholesterol (HDL-C), systolic blood pressure (SBP), diastolic blood pressure (DBP), geographic region, level of urbanization, treatment for hypertension, diabetes, current smoking status, and family history of ASCVD (17). Since all participants in this study were from Shanghai, their place of residence was initially categorized as a southern city. A 10Y-ASCVDR of <10% was defined as the low-risk group (LR group), while a 10Y-ASCVDR of ≥10% was classified as the high-risk group (HR group), which were clinically meaningful cut points (17, 18).

### 2.6 Echocardiography

All participants were placed in the left lateral lying position, breathing steadily, and connected to a synchronous ECG using a Philips EPIQ7 echocardiography system with an S5-1 probe and a frame rate of ≥60 frames/min. Left ventricular end-diastolic/systolic anteroposterior diameter (LVEDD/LVESD), left ventricular end-diastolic septal thickness (IVSd), and left ventricular end-diastolic posterior wall thickness (LVPWd) were measured. Left ventricular mass was calculated according to the Devereux formula = 0.8 × 1.04[(LVEDD + IVSd + LVPWd)^3^ − LVEDD^3^] + 0.6 and was indexed to the body surface area (19). Relative wall thickness (RWT) was calculated using the following formula: RWT = [2 × LVPWd/LVEDD)]. According to guidelines, LV geometric changes were assessed and classified into four patterns: normal, concentric remodeling, concentric, or eccentric hypertrophy (20). Pulse Doppler measurements were performed to determine mitral flow velocity (E and A waves), and tissue Doppler imaging was performed to assess septal and lateral mitral annular early myocardial relaxation velocity from apical four-chamber views. LVEF was measured using the biplane Simpson's method (21). Two-dimensional grayscale images of three consecutive cardiac cycles were acquired and stored at end-expiration for offline analysis. During our study, chamber quantification parameters were measured and calculated based on recommendations from the 2015 American Society of Echocardiography/European Association of Cardiovascular Imaging (20). Echocardiographic acquisitions and 2D-STE analyses were performed by two cardiologists with extensive experience.

The diagnostic algorithm for heart failure with preserved ejection fraction (HFpEF), as proposed in the 2019 consensus recommendations of the Heart Failure Association of the European Society of Cardiology (22), was applied to the patients in this study. HFpEF was determined based on the presence of signs and symptoms of heart failure, echocardiographic findings, and elevated natriuretic peptide levels.

### 2.7 Speckle-tracking image analysis

Utilizing 2D-STE software (QLAB13.0, Philips Healthcare), parasternal short-axis and long-axis views of three consecutive cardiac cycles were analyzed. Our sketch of the subendocardium was based on the apical four-chamber, three-chamber, and two-chamber views, as well as short-axis views at the mitral valve, papillary, and apical levels, respectively, and confirmed the aortic valve closure time in the apical three-chamber view. The 2D-STE software was then used to automatically create a region of interest that contained subendocardial, mid-, and subepicardial regions, adjusting the region to adequately include the myocardium without including the pericardium. By carefully inspecting the endocardial border, the region of interest was adjusted, and if necessary, manual corrections were made. If, after manual adjustments, a participant had more than one under-tracked segment in at least one apical or short-axis view, the LV GLS or GCS data were not used for further data analysis. Apical views were analyzed to obtain the longitudinal strain, while short-axis views were used to calculate the circumferential strain.

**Figure 1:**
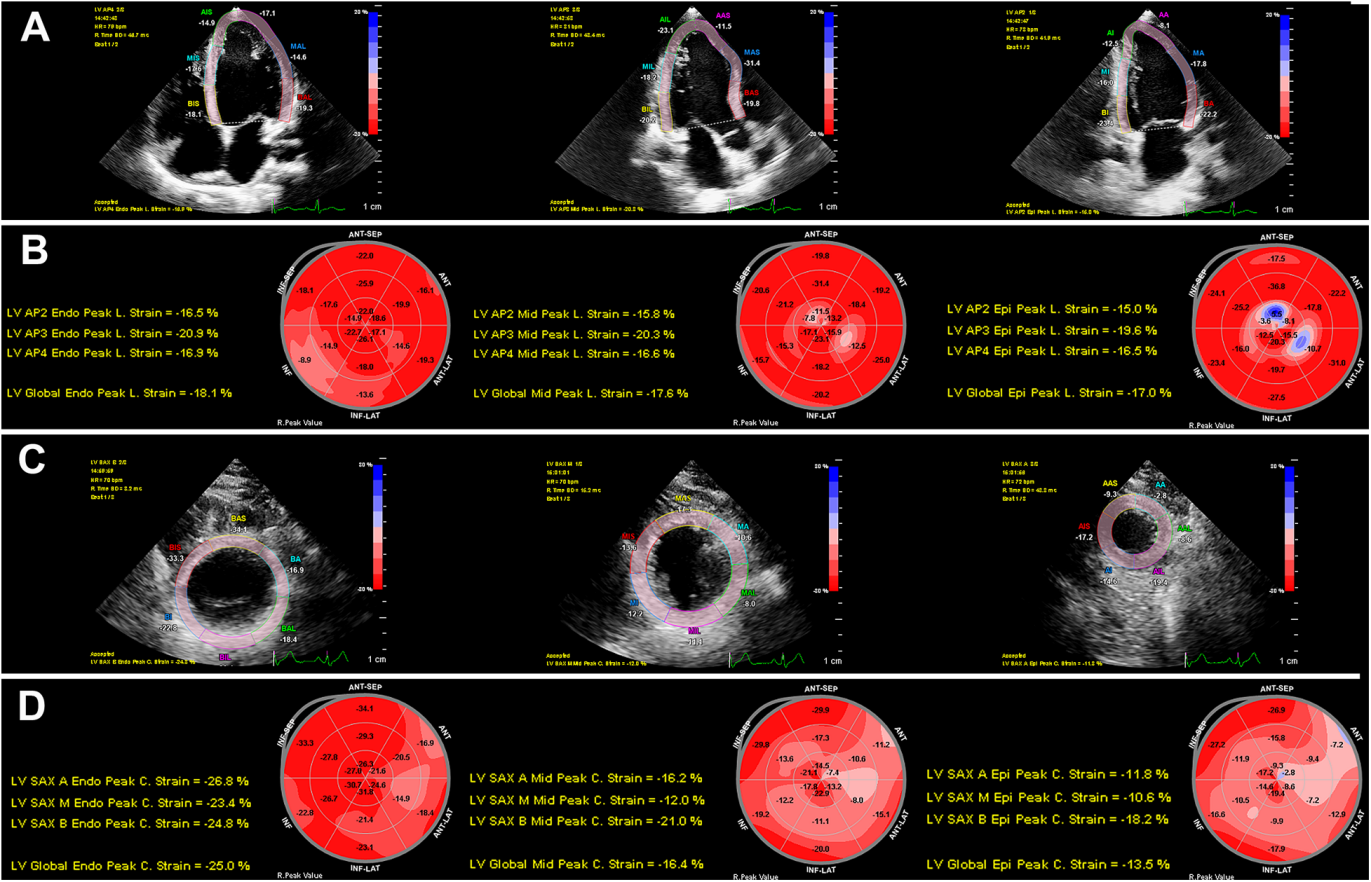
Two-dimensional speckle-tracking analyses of myocardial layer-specific strain. **(A)** LV endocardial tracing in the LV apical four-chamber, three-chamber, and two-chamber views. **(B)** 18-segment bull's eye diagram of GLS_endo_, GLS_mid_, and GLS_epi_. **(C)** LV endocardial tracing in the LV short-axis views at the basal, middle, and apical levels. **(D)** 18-segment bull's eye diagram of GCS_endo_, GCS_mid_, and GCS_epi_. GCS, global circumferential strain; GLS, global longitudinal strain; LV, left ventricular.

In healthy individuals, late systolic shortening (following aortic valve closure) may occur; therefore, peak systolic strain before aortic valve closure and maximum strain value of the entire cardiac cycle were calculated globally for the GLS_ww_, endomyocardial global longitudinal strain (GLS_endo_), midmyocardial global longitudinal strain (GLS_mid_), and GLS_epi_ (23). Mean systolic and maximal values were calculated for each of the six regional segments. The same semi-automated 2D-STE analysis was performed in the parasternal short-axis view at the level of the papillary muscles. We calculated the maximum circumferential strain and the peak systolic strain for the whole wall and layer-specific GCS based on the mean of all six segments (24), including whole-wall global circumferential strain (GCS_ww_), endomyocardial global circumferential strain (GCS_endo_), midmyocardial global circumferential strain (GCS_mid_), and epimyocardial global circumferential strain (GCS_epi_) (Figure 1). Inter- and intra-observer variability was measured in 20 randomly selected participants. The clinical data were blinded to both investigators. Secondary speckle-tracking analyses were performed over one month after the original analysis. We calculated the absolute bias (mean difference), the limit of agreement (1.96 standard deviation), and intra-class correlations for each parameter.

### 2.8 Blood biochemical analysis

Participants were instructed to fast for more than 12 h, after which 5 ml of venous blood was drawn the following day. Relevant biochemical indices, including TC, HDL-C, low-density lipoprotein cholesterol (LDL-C), triglyceride (TG), and glucose levels, were measured using immunoturbidimetry with an automated biochemical instrument. In addition, renal function was assessed by measuring blood creatinine levels, and creatinine clearance was calculated using the Modification of Diet in Renal Disease (MDRD) formula. Impaired glucose regulation (IGR) was defined using the World Health Organization criteria: fasting plasma glucose between 6.1 and 6.9 mmol/L and/or 2-h glucose between 7.8 and 11.1 mmol/L (25).

### 2.9 Statistical analysis

Data were presented in categorical form as numbers and percentages for qualitative variables, while quantitative variables were expressed as the mean and standard deviation for normally distributed days or as the median (interquartile range) for non-normally distributed continuous variables. Statistical analysis was performed using IBM SPSS software, version 23.0. The Student's *t*-test was employed to compare normally distributed variables between groups, and the Wilcoxon rank-sum test was used for variables with skewed distributions. The chi-square test or Fisher's exact test was applied to analyze categorical variables. One-way ANOVA and the Bonferroni test were used for inter- and intra-group comparisons of continuous data.

Pearson's or Spearman’s correlation analysis was used to examine the relationships between layer-specific GLS, layer-specific GCS, and all other variables. To identify risk factors associated with hypertension, multivariate logistic regression analysis was conducted, including clinical data such as BMI, SBP, DBP, and HDL-C.

The relationship between myocardial layer-specific strain and 10Y-ASCVDR was analyzed by restrictive cubic spline (RCS) at the 0th, 5th, 27.5th, 50th, 77.5th, 95th, and 100th percentiles. Statistical analysis of the RCS was performed using R version 4.2.2 (R Foundation for Statistical Computing, Vienna, Austria). To further analyze the effect of myocardial layer-specific strain on high 10Y-ASCVDR, backward stepwise regression methods were used to determine the features associated with high 10Y-ASCVDR. Three models (one clinical and two echocardiographic) were created to avoid overfitting. Variables included in the analysis were clinical data, such as age, sex, hypertension, current smoking status, chronic kidney disease, HFpEF, dyslipidemia, and impaired glucose regulation, and echocardiographic data, such as GLS and GCS. A *p*-value <0.05 was considered statistically significant.

## 3 Results

### 3.1 Baseline characteristics

Fourteen patients were excluded because of poor echocardiographic image quality for strain analysis. Finally, 239 hypertensive patients (average age 59 years, 56.1% male) and 124 control participants (average age 57 years, 46.8% male) were included in the analysis.

The most commonly used antihypertensive medications in the overall population were calcium channel blockers (28.9%) and angiotensin receptor blockers (28.0%). The BMI, SBP, and DBP of the subjects in the hypertensive group were higher than those in the control group (*p <* 0.05), while the HDL-C levels of the subjects in the hypertensive group were lower than those in the control group (*p <* 0.01). Compared with the control group, 10Y-ASCVDR in the hypertensive group was higher (*p <* 0.05) as was the proportion of individuals with high 10Y-ASCVDR (*p <* 0.05) (Table 1). Left ventricular structural parameters, including LVEDD, IVSd, LVPWd, PWT, LV mass index, and E/e’, were higher in the hypertensive group than in the control group (*p <* 0.05). The absolute values of GLS_ww_, GLS_endo_, GLS_mid_, and GLS_epi_ were lower in the hypertensive group than in the control group (*p <* 0.05). Compared to the control group, hypertensive patients experienced greater concentric hypertrophy in the LV (Table 2).

**Table 1:** Baseline characteristics of the 363 participants.

Variables	Control group (*n =* 124)	Hypertensive group (*n =* 239)	*t/Z/χ^2^*	*p-*value
Age (years)	57.31 ± 12.62	59.42 ± 12.37	−1.535	0.126
Sex (men)	58 (46.8%)	134 (56.1%)	2.829	0.092
Current smoker (*n*)	21 (16.9%)	52 (21.8%)	1.181	0.277
Antihypertension (*n*)	0 (0)	137 (57.3%)		<0.001^a^
ACEI	0 (0)	6 (2.5%)		<0.001^a^
ARB	0 (0)	67 (28.0%)		<0.001^a^
β-blockers	0 (0)	29 (12.1%)		<0.001^a^
Calcium channel blockers	0 (0)	69 (28.9%)		<0.001^a^
Diuretics (loop or thiazide)	0 (0)	11 (4.6%)		<0.001^a^
Comorbidities				
Chronic kidney disease	3 (2.4%)	5 (3.8%)	0.463	0.496
HFpEF	5 (4.0%)	11 (6.3%)	0.790	0.374
Dyslipidemia	20 (16.1%)	51 (21.4%)	1.452	0.228
Impaired glucose regulation	34 (27.4%)	74 (31.0%)	0.490	0.483
Anthropometrics				
BMI (kg/m^2^)	23.00 ± 3.31	24.5 ± 3.69	−3.821	<0.001
SBP (mmHg)	122.12 ± 11.29	150.28 ± 22.14	−13.289	<0.001
DBP (mmHg)	75.94 ± 8.13	90.56 ± 14.26	−10.566	<0.001
Heart rate (beats/min)	77.98 ± 12.56	78.26 ± 13.65	−0.196	0.845
Laboratory parameters				
Total cholesterol (mmol/L)	4.55 ± 1.02	4.64 ± 1.00	−0.810	0.418
HDL-C (mmol/L)	1.19 ± 0.33	1.09 ± 0.27	3.174	0.002
Glucose (mmol/L)	5.85 ± 1.97	6.25 ± 1.93	−1.792	0.074
eGFR (ml/min)	116.04 ± 32.56	112.26 ± 30.01	1.000	0.318
HbA1c (%)	5.66 ± 0.71	5.87 ± 0.54	−1.893	0.060
BNP (pg/ml)	34.50 (17.60–49.25)	27.65 (14.47–48.12)	−0.576	0.564
cTnI (μg/L)	0.00 (0.00–0.00)	0.00 (0.00–0.01)	−1.847	0.065
China-PAR ASCVD risk				
10Y-ASCVDR	5.81 (2.57–10.48)	9.57 (5.74–16.29)	−5.686	<0.001
10Y-ASCVDR ≥10%	36 (29%)	115 (48.1%)	12.240	<0.001

ACEI, angiotensin-converting enzyme inhibitors; ARB, angiotensin receptor blockers; HFpEF, heart failure with preserved ejection fraction; BMI, body mass index; eGFR, estimated glomerular filtration rate; HDL-C, high-density lipoprotein cholesterol; HbA1c, hemoglobin A1c; BNP, brain natriuretic peptide; cTnI, cardiac troponin I; 10Y-ASCVDR, 10-year atherosclerotic cardiovascular disease risk.

Data are expressed as mean ± standard deviation, median (interquartile range), or *n* (%).

^a^ Fisher’s exact test. 1 mmHg = 0.133 kPa.

**Table 2:** Echocardiographic characteristics of the study population.

Variables	Control group (*n =* 124)	Hypertensive group (*n =* 239)	*t/Z/χ^2^*	*p-*value
Echocardiographic characteristics				
LVEDD (mm)	45.09 ± 4.12	46.28 ± 3.63	−2.831	0.005
LVESD (mm)	28.16 ± 3.35	28.80 ± 2.92	−1.887	0.060
IVSd (mm)	9.11 ± 0.98	9.86 ± 1.16	−6.141	<0.001
LVPWd (mm)	8.70 ± 0.86	9.47 ± 1.07	−6.950	<0.001
RWT (cm)	0.39 ± 0.04	0.41 ± 0.05	−4.226	<0.001
LV mass index (g/m^2^)	80.38 ± 14.13	90.3 ± 15.92	−5.844	<0.001
LVEF (%)	67.47 ± 5.25	67.41 ± 4.94	0.110	0.913
E/e’	7.15 ± 2.30	7.94 ± 3.05	−2.526	0.012
GLS_ww_ (%)	−21.30 ± 2.85	−19.99 ± 3.21	−3.838	<0.001
GLS_endo_ (%)	−22.23 ± 3.00	−20.73 ± 3.31	−4.215	<0.001
GLS_mid_ (%)	−21.32 ± 2.88	−19.92 ± 3.27	−4.018	<0.001
GLS_epi_ (%)	−20.35 ± 2.88	−19.32 ± 3.17	−3.044	0.003
GCS_ww_ (%)	−24.28 ± 6.24	−24.06 ± 5.74	−0.318	0.750
GCS_endo_ (%)	−28.16 ± 6.69	−28.05 ± 6.35	−0.151	0.880
GCS_mid_ (%)	−23.74 ± 6.10	−23.48 ± 5.86	−0.383	0.702
GCS_epi_ (%)	−20.94 ± 6.64	−20.51 ± 5.96	−0.609	0.543
Ventricular geometry				
Normal (*n*)	85 (68.5%)	136 (56.9%)	4.648	0.031
Concentric remodeling (*n*)	28 (22.6%)	64 (26.8%)	0.760	0.383
Eccentric hypertrophy (*n*)	11 (8.9%)	15 (6.3%)	0.827	0.363
Concentric hypertrophy (*n*)	0 (0)	24 (10%)		<0.001*

LVEDD, left ventricular end-diastolic diameter; LVESD, left ventricular end-systolic diameter; IVSd, interventricular septum diameter; LVPWd, left ventricular end-diastolic posterior wall thickness; RWT, relative wall thickness; LVEF, left ventricular ejection fraction; E, early diastolic mitral flow (pulsed Doppler); e, average of the peak early diastolic relaxation velocity of the septal and lateral mitral annulus (tissue Doppler); GLS_ww_, whole-wall global longitudinal strain; GLS_endo_, endomyocardial global longitudinal strain; GLS_mid_, midmyocardial global longitudinal strain; GLS_epi_, epimyocardial global longitudinal strain; GCS_ww_, whole-wall global circumferential strain; GCS_endo_, endomyocardial global circumferential strain; GLS_mid_, midmyocardial global circumferential strain; GCS_epi_, epimyocardial global circumferential strain.

* Fisher's exact test.

### 3.2 Reproducibility

Intra- and inter-observer variability was assessed for all global strain parameters. Bias, limits of agreement, and intra-class correlation coefficients (ICCs) for GLS_ww_, GLS_endo_, GLS_mid_, GLS_epi_, GCS_ww_, GCS_endo_, GCS_mid_, and GCS_epi_ are reported in Table 3. The intra- and inter-observer ICCs were 0.941 and 0.848 for GLS_ww_, 0.943 and 0.858 for GLS_endo_, 0.964 and 0.852 for GLS_mid_, 0.940 and 0.833 for GLS_epi_, 0.955 and 0.879 for GCS_ww_, 0.944 and 0.864 for GCS_endo_, 0.950 and 0.888 for GCS_mid_, and 0.961 and 0.885 for GCS_epi_, respectively.

**Table 3:** Intra- and inter-observer variability.

	Intra-observer variability			Inter-observer variability		
	Mean difference	1.96 SD	ICC	Mean difference	1.96 SD	ICC
GLS_ww_	0.52	4.39	0.941	0.56	7.44	0.848
GLS_endo_	0.44	4.75	0.943	0.50	7.74	0.858
GLS_mid_	0.65	4.64	0.940	0.71	7.12	0.852
GLS_epi_	0.47	4.42	0.940	0.49	7.86	0.833
GCS_ww_	0.44	4.44	0.955	0.47	7.56	0.879
GCS_endo_	0.39	4.38	0.944	0.45	7.26	0.864
GCS_mid_	0.49	4.51	0.950	0.55	7.41	0.888
GCS_epi_	0.46	4.48	0.961	0.42	8.05	0.885

ICC, intra-class correlation coefficient; SD, standard deviation.

### 3.3 Relationship between layer-specific GLS, GCS, and hypertension

Because clinical parameters, such as BMI, HDL-C, and blood pressure, were significantly different between the two groups, we performed a multiple logistic regression analysis to identify differences in myocardial strain associated with hypertension. GLS_ww,_ GLS_endo_, and GLS_mid_ were independently associated with hypertension, with odds ratios (ORs) of 1.131, 1.154, and 1.133, respectively (*p =* 0.031, 0.007, and 0.026; Table 4).

**Table 4:** Logistic regression analysis of myocardial layer-specific strain associated with hypertension.

Variable	Standardized *β-*coefficient	OR (95% CI)	*p-*value
Model 1			
GCS_ww_	−0.043	0.958 (0.904–1.016)	0.152
GLS_ww_	0.123	1.131 (1.011–1.264)	0.031
Model 2			
GCS_endo_	−0.041	0.960 (0.910–1.012)	0.129
GLS_endo_	0.143	1.154 (1.039–1.281)	0.007
Model 3			
GCS_mid_	−0.039	0.961 (0.907–1.019)	0.182
GLS_mid_	0.125	1.133 (1.015–1.265)	0.026
Model 4			
GCS_epi_	−0.029	0.971 (0.92–1.025)	0.285
GLS_epi_	0.096	1.101 (0.987–1.229)	0.085

OR, odds ratio; CI, confidence interval.

Using logistic regression analysis while adjusting for the effects of variables including BMI, SBP, DBP, and HDL-C in multivariable models 1–4.

### 3.4 Relationship between layer-specific GLS, GCS, and clinical variables

**Figure 2:**
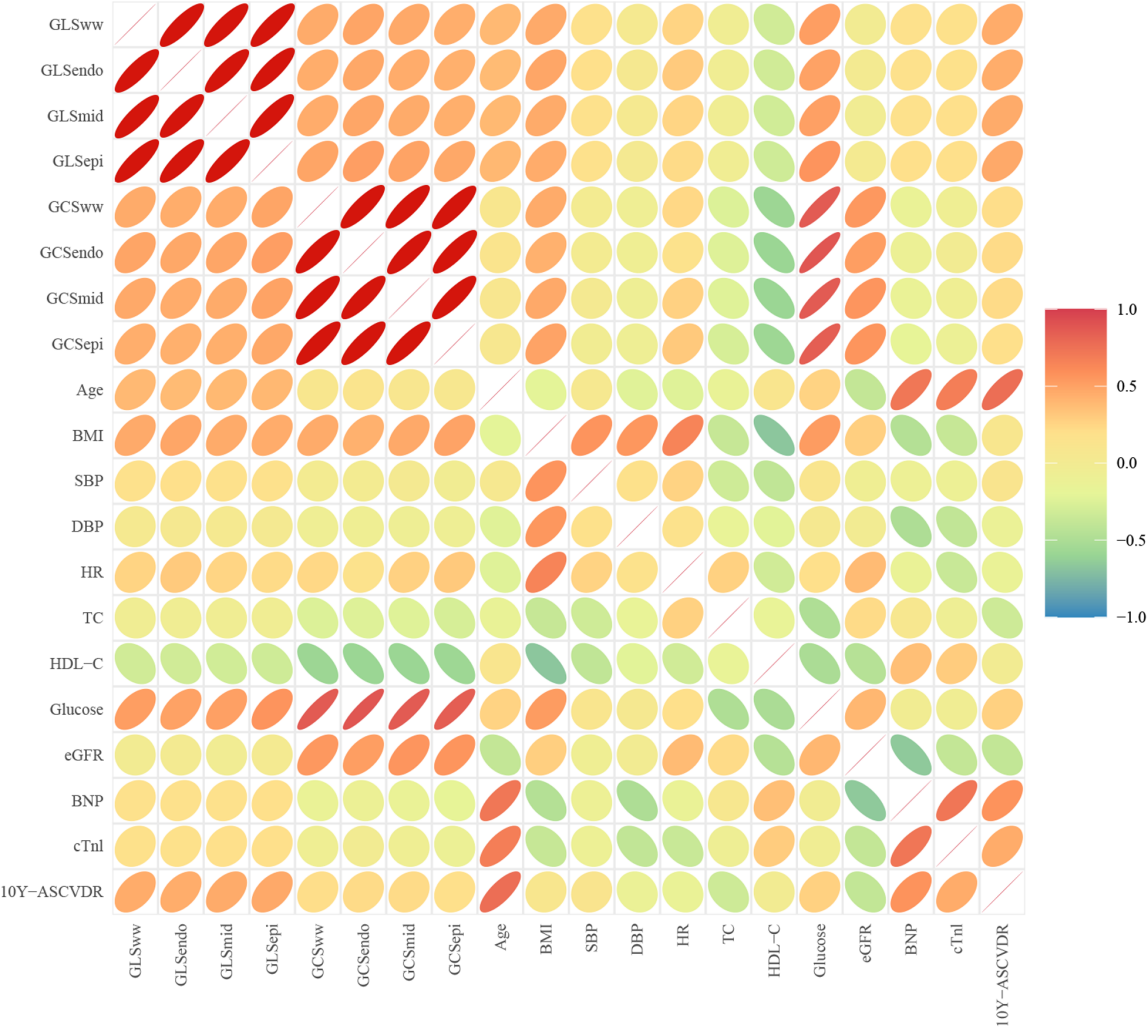
Correlation between clinical parameters and myocardial layer-specific strain. Positive correlations were found between age and layer-specific GLS (*p <* 0.05) and layer-specific GLS, GCS, and glucose (*p <* 0.05). Negative correlations were observed between layer-specific GCS, GLS, and HDL-C (*p <* 0.05).

Positive correlations were found between age and GLS [GLS_ww_, GLS_endo_, GLS_mid_, and GLS_epi_ (*r* = 0.20, 0.17, 0.20, and 0.23)], glucose and GLS [GLS_ww_, GLS_endo_, GLS_mid_, and GLS_epi_ (*r* = 0.22, 0.21, 0.22, and 0.21)], and glucose and GCS [GCS_ww_, GCS_endo_, GCS_mid_, and GCS_epi_ (*r* = 0.34, 0.32, 0.34, and 0.31)]. Negative correlations were observed between HDL-C and GLS [GLS_ww_, GLS_endo_, GLS_mid_, and GLS_epi_ (*r* = −0.18, −0.19, −0.17, and −0.16)] and HDL-C and GCS [GCS_ww_, GCS_endo_, GCS_mid_, and GCS_epi_ (*r* = −0.17, −0.15, −0.20, and −0.14)] (Figure 2).

### 3.5 Comparison of myocardial layer-specific strain in different cardiovascular risks

**Figure 3:**
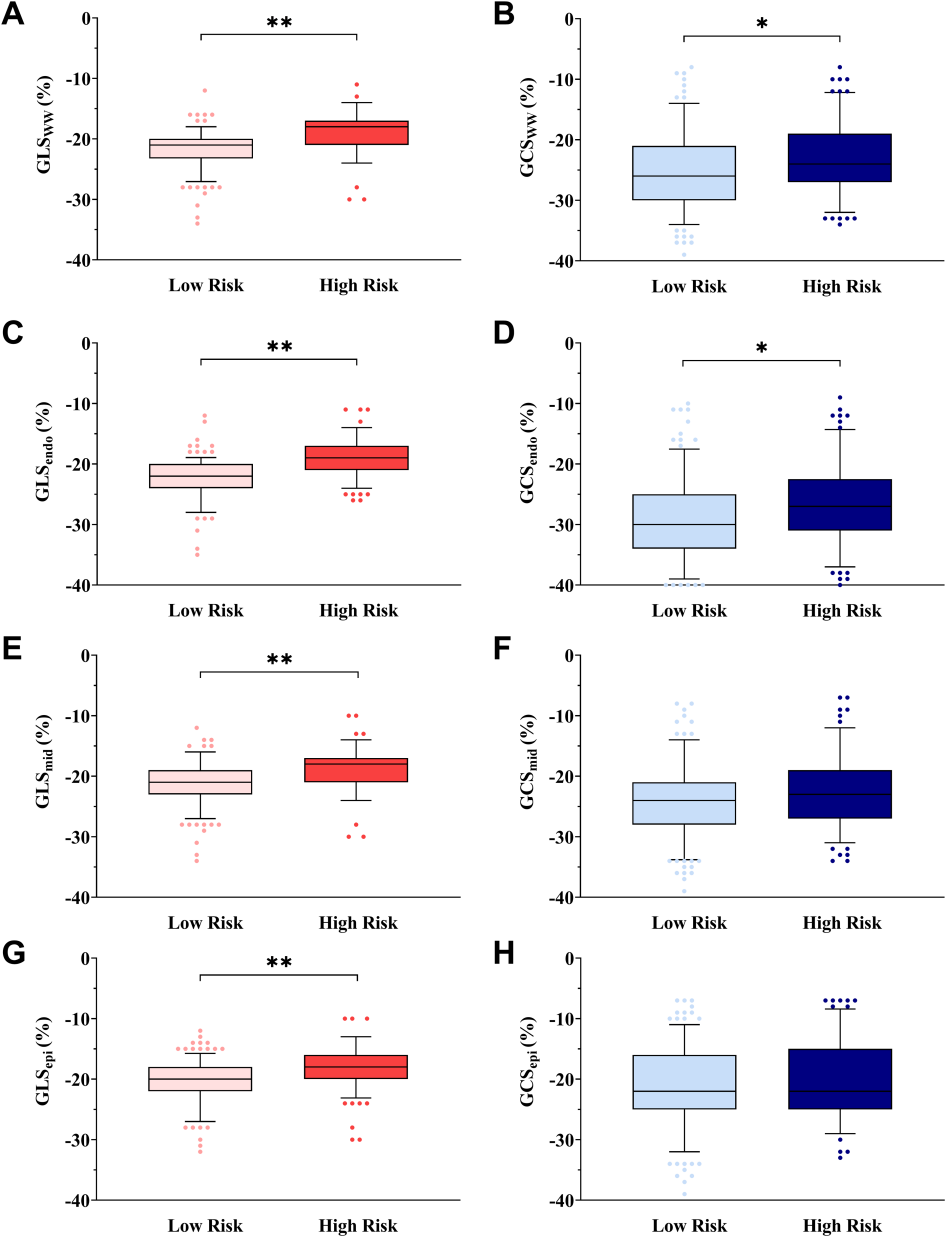
Box-and-whisker plot showing myocardial layer-specific strain in cardiovascular risk stratification. The box-and-whisker plot showed that the absolute values of GLS_ww_ **(A)**, GCS_ww_ **(B)**, GLS_endo_ **(C)**, GCS_endo_ **(D)**, GLS_mid_ **(E)**, and GLS_epi_ **(G)** were lower in the HR group than in the LR group in all subjects, and GCS_mid_ **(F)** or GCS_epi_ **(H)** was not statistically different in all subjects. Low risk is defined as 10Y-ASCVDR <10%. High risk is defined as 10Y-ASCVDR ≥10%. *p < 0.05, **p < 0.01, ANOVA followed by a *post-hoc Q*-test method.

The absolute values of GLS_ww_, GLS_endo_, GLS_mid_, and GLS_epi_ were lower in the HR group than in the LR group across all participants (*p <* 0.05), while GCS_mid_ and GCS_epi_ were not statistically different among the four groups (*p* > 0.05). The absolute values of GCS_ww_ and GCS_endo_ were lower in the HR group than in the LR group within both the overall population and the control group (*p <* 0.05) (Figure 3).

### 3.6 Relationship between myocardial layer-specific strain and 10Y-ASCVDR

**Figure 4:**
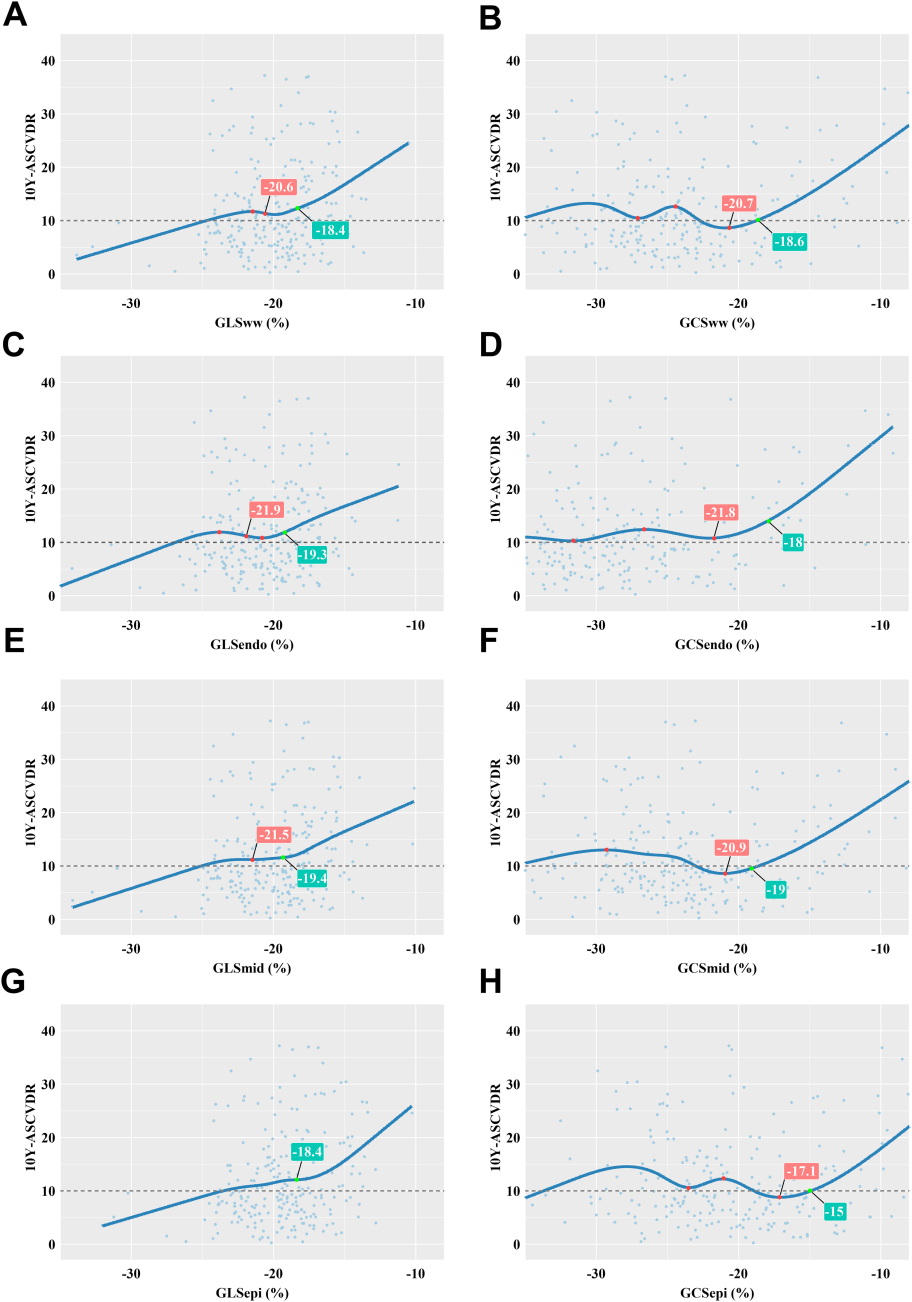
Correlation between myocardial layer-specific strain and 10Y-ASCVDR based on a restricted cubic spline function. **(A)** 10Y-ASCVDR increased rapidly as the GLS_ww_ increased, until it slowed at a GLS_ww_ of −20.6% and then increased rapidly again when the GLS_ww_ reached −18.4%. **(B)** There was a significant J-shaped relationship between GCS_ww_ and 10Y-ASCVDR. **(C)** 10Y-ASCVDR increased rapidly as the GLS_endo_ increased, until it slowed down at a GLS_endo_ of −21.9% and then increased rapidly again when the GLS_endo_ reached −19.3%. **(D)** There was a significant J-shaped relationship between GCS_endo_ and 10Y-ASCVDR. **(E)** 10Y-ASCVDR increased rapidly as the GLS_mid_ increased, until it slowed down at a GLS_mid_ of −21.5% and then increased rapidly again when the GLS_mid_ reached −19.4%. **(F)** There was a significant J-shaped relationship between GCS_mid_ and 10Y-ASCVDR. **(G)** 10Y-ASCVDR increased with GLS_epi_, notably accelerating at a GLS_epi_ of −18.4%. (**H**) There was a significant J-shaped relationship between GCS_epi_ and 10Y-ASCVDR.

The relationship between different myocardial layer-specific strains and 10Y-ASCVDR was graphically depicted using a restrictive cubic spline for analysis. The analysis unveiled a significant J-shaped relationship between GCS and 10Y-ASCVDR. For the reduced absolute value of GLS, there were increases in 10Y-ASCVDR, with a rapid increase observed at a GLS_ww_ value of −18.4%, GLS_endo_ value of −19.3%, GLS_mid_ value of −19.4%, or GLS_epi_ value of −18.4%, as illustrated in Figure 4.

The logistic regression model showed that multiple factors, including sex, age, hypertension, impaired glucose regulation, smoking status, HDL-C, TC, SBP, GLS, and GCS, were independent variables that significantly affected high 10Y-ASCVDR; these variables revealed significantly different associations with myocardial stratified strain. After adjusting for confounding factors, we found that GLS_ww_ was an independent risk factor for high 10Y-ASCVDR [OR 1.169, 95% confidence interval (CI) 1.006–1.359, *p* = 0.042], whereas GCS_ww_ was not. In further strain analysis of different myocardial layers, we found that GLS_mid_ was an independent risk factor for high 10Y-ASCVDR (OR 1.175, 95% CI 1.014–1.362, *p* = 0.032), as was GCS_endo_ (OR 1.177, 95% CI 1.022–1.356, *p* = 0.024). On the contrary, GCS_mid_ was identified as an independent protective factor against high 10Y-ASCVDR (OR 0.84, 95% CI 0.717–0.983, *p* = 0.03) (Table 5).

**Table 5:** Logistic regression analysis of myocardial layer-specific strain associated with high 10Y-ASCVDR (step).

Variable	Standardized *β*-coefficient	OR (95% CI)	*p-*value
Model 1^a^			
Sex	2.946	19.022 (7.237–49.998)	<0.001
Age (years)	0.304	1.355 (1.259–1.459)	<0.001
Hypertension	1.497	4.466 (1.849–10.787)	0.001
Impaired glucose regulation	2.300	9.979 (3.394–29.338)	<0.001
Current smoker	1.721	5.587 (1.708–18.279)	0.004
GLS_ww_ (%)	−0.162	0.85 (0.732–0.988)	0.034
Model 2^b^			
Sex	2.611	13.617 (5.460–33.961)	<0.001
Age (years)	0.300	1.35 (1.259–1.447)	<0.001
HDL-C (mmol/L)	−0.085	0.918 (0.877–0.962)	<0.001
Total cholesterol (mmol/L)	0.013	1.013 (1.002–1.023)	0.020
SBP (mmHg)	0.042	1.043 (1.024–1.063)	<0.001
GLS_ww_ (%)	0.156	1.169 (1.006–1.359)	0.042
GCS_ww_ (%)	0.009	1.009 (0.941–1.081)	0.809
Model 3^c^			
Sex	2.683	14.622 (5.745–37.215)	<0.001
Age (years)	0.303	1.353 (1.260–1.453)	<0.001
HDL-C (mmol/L)	−0.092	0.912 (0.869–0.957)	<0.001
Total cholesterol (mmol/L)	0.013	1.013 (1.002–1.024)	0.019
SBP (mmHg)	0.044	1.045 (1.025–1.065)	<0.001
GLS_mid_ (%)	0.161	1.175 (1.014–1.362)	0.032
GCS_endo_ (%)	0.163	1.177 (1.022–1.356)	0.024
GCS_mid_ (%)	−0.175	0.840 (0.717–0.983)	0.030

^a^ Multivariable model 1 included the following: age, sex, hypertension, current smoker, chronic kidney disease, HFpEF, dyslipidemia, impaired glucose regulation, GLS_ww_, and GCS_ww_.

^b^ Multivariable model 2 included the following: age, sex, BMI, HDL-C, total cholesterol, systolic and diastolic blood pressure, GLS_ww_, and GCS_ww_.

^c^ Multivariable model 3 included the following: model 2 and GLS_endo_, GLS_mid_, GLS_epi_, GCS_endo_, GLS_mid_, and GCS_epi_.

## 4 Discussion

In this study, we observed an association between hypertensive patients with high 10Y-ASCVDR and impaired systolic cardiac function. The 10Y-ASCVDR values exhibited a significant J-shaped relationship with layer-specific GLS and GCS. GLS_ww_, GLS_mid_, and GCS_endo_ were identified as independent influencing factors of high 10Y-ASCVDR. However, it is worth noting that GCS_mid_ was found to be a protective factor against high 10Y-ASCVDR.

In this study, the values of GLS_ww_, GLS_epi_, GLS_mid_, and GLS_endo_ were significantly lower in hypertensive patients with high-risk ASCVDR than in hypertensive patients with low-risk ASCVDR and controls. In addition, GLS_ww_ was found to be an effective predictor of 10Y-ASCVDR. Saito et al. (26) followed 388 asymptomatic non-ischemic patients with hypertension for 10 years and identified GLS as an independent predictor of 10Y-ASCVDR. Abnormal myocardial morphology precedes clinical symptoms, and observation of myocardial strain allows early assessment of impaired cardiac function and regional differences in myocardial performance (27). Left ventricular GLS and GCS are sensitive indicators for assessing early changes in myocardial systolic function in cardiovascular disease and can be used to diagnose microvascular disease (28, 29). In our study, we found that the layer-specific GLS of hypertensive patients with normal LVEF had already started to decline, while layer-specific GCS did not show significant changes, indicating that reduced longitudinal myocardial strain precedes circumferential myocardial strain and may occur before changes in overall LV systolic function. Previous studies have found that GLS is highly sensitive to myocardial injury and may reflect cardiac contractile function, while GCS has high specificity and may reflect changes in cardiac configuration (29, 30). Longitudinal left ventricular fibers are more susceptible to elevated blood pressure due to their anatomy and more sensitive to myocardial interstitial fibrosis, which may lead to microcirculatory disturbances and affect their systolic function (31, 32). Interestingly, our study showed a significant J-shaped association between GCS and China-PAR scores. We speculate that this result may be a manifestation of compensatory function in GCS, as GCS has high specificity and may reflect changes in cardiac configuration. Severe damage to GCS may cause irreversible damage to the heart. In the early stages of myocardial injury, the circumferential myocardial fibers maintain cardiac function rather than the more vulnerable longitudinal subendocardial fibers (29). If the patient experiences decompensated circumferential myocardial diastolic function, this may indicate the occurrence of cardiovascular events (33).

In the early stages of myocardial injury, the human heart exhibits heterogeneity in its transmural contraction characteristics (34). Our study found a decrease in the absolute value of GCS_endo_ in high-risk populations, which further proved that the subendocardial myocardium is more susceptible to damage within the three-layered myocardium. This may be because the subendocardial fibers in the three-layered myocardium are most susceptible to ischemia, hypoperfusion, and fibrosis due to aging (35). We found an interesting result that GCS_mid_ was a protective factor against high 10Y-ASCVDR, indicating that GCS_mid_ may increase to compensate for the worsening of GCS_endo_. Recent studies have also identified that GLS_mid_ is a stronger predictor of cardiovascular events than GLS_endo_ in patients with hypertrophic cardiovascular disease (36). Haynes et al. (34) found results consistent with our study, indicating that the non-failing mid-myocardium develops more power output than the endocardium and epicardium, while the failing mid-myocardium develops less power output due to increased fibrosis, which is associated with elevated levels of cardiac troponin I, fibronectin, and myosin light chain-1. The coronary arteries supply blood vertically to the myocardium, from the outer layer to the inner layer, making the subendocardial myocardium the most sensitive to ischemia (37). As the degree of myocardial ischemia increases, the middle and even outer layers of the myocardium also show functional damage, indicating that myocardial layer-specific strain can more accurately evaluate the degree of myocardial ischemia at three different layers of muscle fibers (37). Circumferential strain remains normal in hypertensive patients with normal LVEF, indicating that circumferential strain plays a vital role in maintaining LVEF as the left ventricular myocardium undergoes remodeling and longitudinal strain is reduced (38). In clinical scenarios involving hypertensive heart disease, the short-axis compensatory function is not unlimited. As longitudinal function is already impaired in hypertensive heart disease, the subsequent loss of short-axis reserve may theoretically lead to the development of heart failure (39).

### 4.1 Limitations

This study has several limitations. First, the sample of this study mainly consisted of single-center data from hypertensive patients in Shanghai, China. There was a selection bias between the southern urban population and other populations, which may affect the generalizability of the results. Second, the classification of hypertension was not studied and needs to be further explored. Third, among multidirectional LV myocardial deformations, we analyzed only longitudinal and circumferential strains. However, GLS and GCS are most likely to be impaired in hypertension, as the function of the myocardium is predominantly determined by the longitudinal and circumferential orientation of myofibers. The evaluation of radial strain in each myocardial layer is also expected to show meaningful results in future studies.

## 5 Conclusions

In hypertensive patients, myocardial layer-specific strain, especially GLS, sensitively detected LV dysfunction and showed a significant J-shaped relationship with 10Y-ASCVDR. GCS_mid_ may have a compensatory effect on myocardial impairment. Understanding LV myocardial layer-specific strain may provide insight into the early compensatory mechanisms of the myocardium in hypertension and aid in managing patients with hypertension, thus preventing further damage.

## Data Availability

The raw data supporting the conclusions of this article will be made available by the authors without undue reservation.
